# Supplementary material for: The Modulation of Respiratory Epithelial Cell Differentiation by the Thickness of an Electrospun Poly-ε-Carprolactone Mesh Mimicking the Basement Membrane
Source: Int J Mol Sci. 2024 Jun 17;25(12):6650. doi: 10.3390/ijms25126650 (PMC11203971; doi:10.3390/ijms25126650)
Supplement: Supplementary file 1 [file ijms-25-06650-s001.zip › Choi, SY et al._Supplement_Table S1.pdf]

Table S1. Primer set for quantitative real-time PCR

Target	Accession Number	Sequence (5’ → 3’)
MUC5AC	NM_001304359.2	5’-CACGTCCCCTTCAATATCCA-3’
		5’-GGCCCAGGTCTCACCTTT-3’
MUC5B	NM_002458.3	5’-GTACAATGGCACCTTCTACGG-3’
		5’-CTGACATTGCACCGTTGG-3’
FOXJ1	NM_001454.4	5’-CAGATCCCACCTGGCAGA-3’
		5’-CGTACTGGGGGTCAATGC-3’
SCGB1A1	NM_003357.5	5’-CAAAAGCCCAGAGAAAGCATC-3’
		5’-CAGTTGGGGATCTTCAGCTTC-3’
CK5	NM_000424.4	5’-TTCATGAAGATGTTCTTTGATGC-3’
		5’-AGGTTGCGGTTGTTGTCC-3’
E-cadherin	Z13009.1	5’-CACGGTAACCGATCAGAATG-3’
		5’-ACCTCCATCACAGAGGTTCC-3’
N-cadherin	NM_001308176	5’-AGCCAACCTTAACTGAGGAGT-3’
		5’-GGCAAGTTGATTGGAGGGATG-3’
TWIST	NM_000474.4	5’-TGCATGCATTCTCAAGAGGT-3’
		5’-GTTTTGCAGGCCAGTTTGAT-3’
SNAIL	NM_005985	5’-CTAGGCCCTGGCTGCTAC-3’
		5’-GACATCTGAGTGGGTCTGGA-3’
TGF-β	M60316.1	5’-ACTATTGCTTCAGCTCCACGGA-3’
		5’-AGTCAATGTACAGCTGCCGCA-3’
α-SMA)	NM_001141945	5’-CTTTCTACAATGAGCTTCGTG-3’
		5’-ATTTGAGTCATTTTCTCCCG-3’
FN1	NM_001306129.2	5’-CCGCCGAATGTAGGACAAGA-3’
		5’-GACAGAGTTGCCCACGGTAA-3’
TNF-α	NM_000594.4	5’-TGGAACTGGCAGAAGAGGCACT-3’
		5’-CCATAGAACTGATGAGAGGGAGGC-3’
IL-6	NM_000600	5’-AATTCGGTACATCCTCGACGG-3’
		5’-GTTTGTTTTCTGCCAGTGCCT-3’
RAC1	NM_006908.5	5’-TGATGCAGGCCATCAAGTGT-3’
		5’-AGAACACATCTGTTTGCGGA-3’
NOX1	NM_007052.5	5’-TTGAAAGGTTGGGTTTAGCTG-3’
		5’-AAATGGAACCCTTGGAGCA-3’
NOX2	NM_000397.4	5’-AAGAGAAACTCCTCTGCTGTGAA-3’
		5’-CGCACTGGAACCCCTGAGAAAGG-3’
NOX4	NM_016931.5	5’-CAGATGTTGGGGCTAGGATT-3’
		5’-AGAAGTTGAGGGCATTCACC-3’
GAPDH	NM_001256799.3	5’-ACCACCTGGTCTCCTCTGAC-3’
		5’-TGCTGTAGCCAAATTCGTTG-3’
