# Supplementary figures and images for: The Modulation of Respiratory Epithelial Cell Differentiation by the Thickness of an Electrospun Poly-ε-Carprolactone Mesh Mimicking the Basement Membrane
Source: Int J Mol Sci. 2024 Jun 17;25(12):6650. doi: 10.3390/ijms25126650 (PMC11203971; doi:10.3390/ijms25126650)

Figure S1.

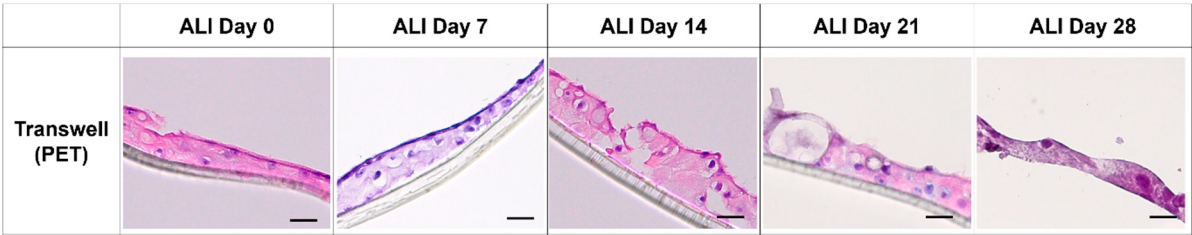

Supplement: Supplementary file 1 [file ijms-25-06650-s001.zip › Choi, SY et al._Supplement_Figure S1.pdf]

**Figure S2.**

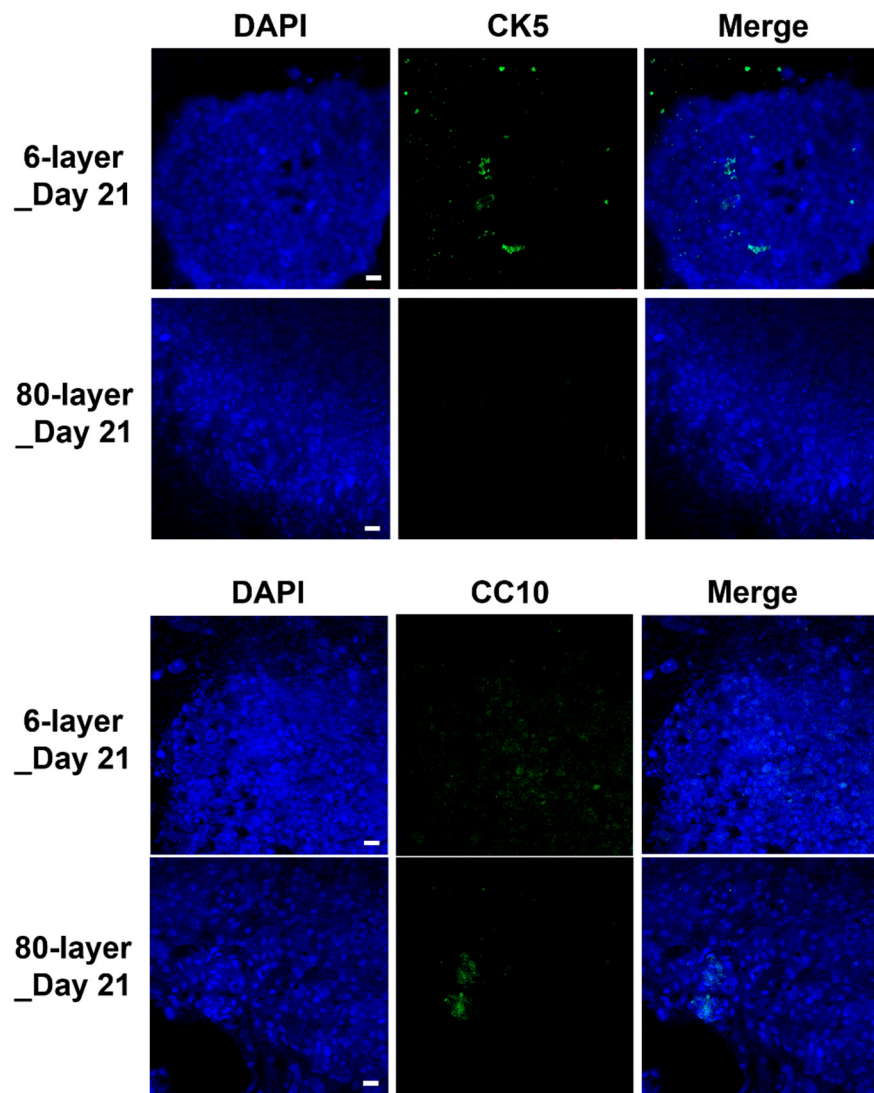

Supplement: Supplementary file 1 [file ijms-25-06650-s001.zip › Choi, SY et al._Supplement_Figure S2.pdf]

**Figure S3.**

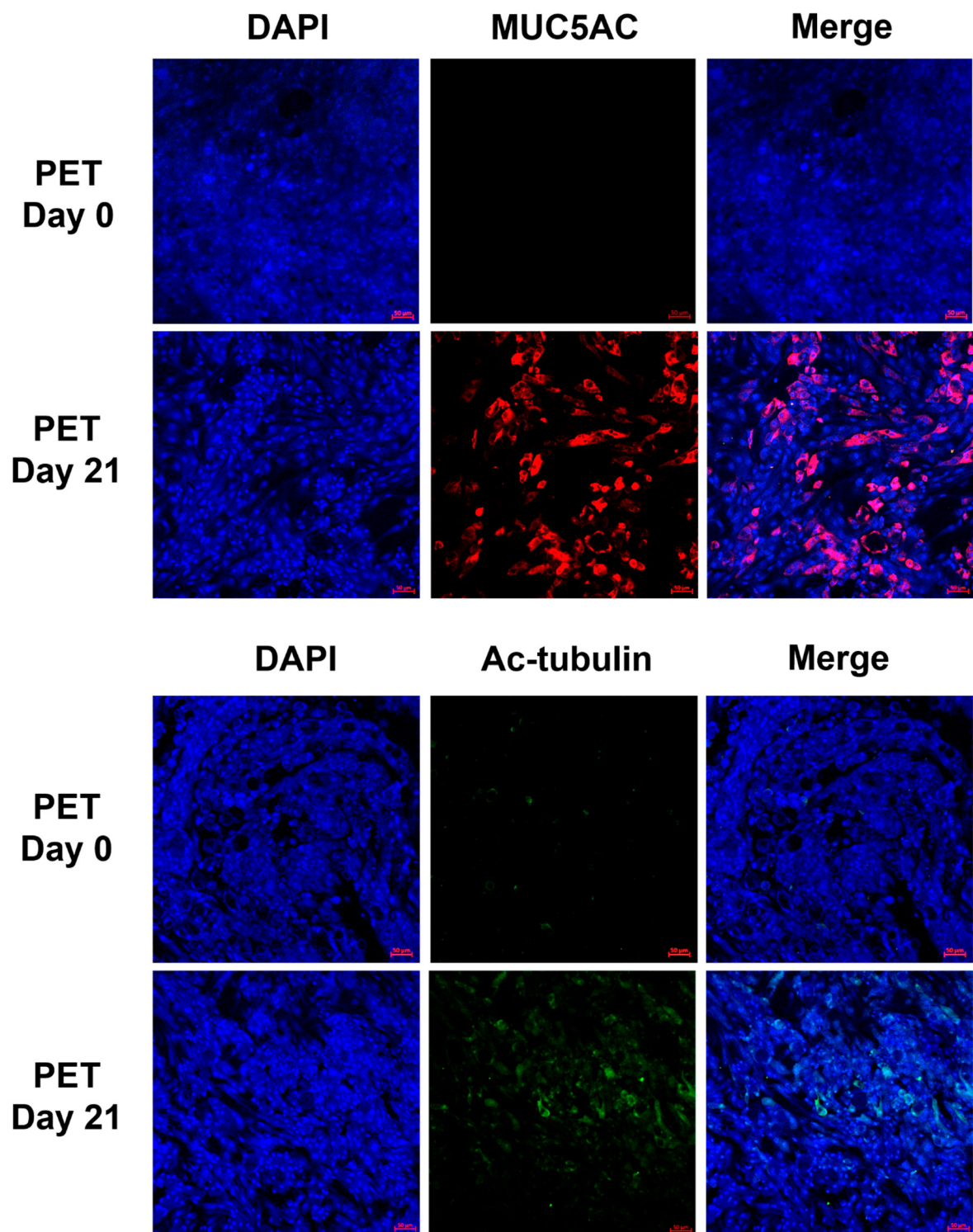

Supplement: Supplementary file 1 [file ijms-25-06650-s001.zip › Choi, SY et al._Supplement_Figure S3.pdf]
